# Antioxidant-Rich Diet, *GSTP1* rs1871042 Polymorphism, and Gastric Cancer Risk in a Hospital-Based Case-Control Study

**DOI:** 10.3389/fonc.2020.596355

**Published:** 2021-01-29

**Authors:** Jimi Kim, Hyejin Kim, Jeonghee Lee, Il Ju Choi, Young-Il Kim, Jeongseon Kim

**Affiliations:** ^1^ Department of Cancer Biomedical Science, Graduate School of Cancer Science and Policy, National Cancer Center, Goyang-si, South Korea; ^2^ Center for Gastric Cancer, National Cancer Center Hospital, National Cancer Center, Goyang-si, South Korea

**Keywords:** gastric cancer, oxygen radical absorbance capacity, glutathione S-transferase Pi, oxidative stress, antioxidants

## Abstract

**Background:**

Chronic gastritis along with *Helicobacter pylori* (*H. pylori*) infection has been implicated in inflammatory response-related genes linked to the causation of gastric cancer. Glutathione S-transferase Pi (GSTP1) plays a role in regulating oxidative stress and detoxification against carcinogenesis. In this study, we aimed to determine whether an antioxidant-rich diet is associated with gastric cancer risk and identify how this association could be altered by *GSTP1* genetic variants.

**Methods:**

This study included 1,245 participants (415 cases and 830 controls) matched for age and sex. The dietary antioxidant capacity was estimated based on the oxygen radical absorbance capacity (ORAC) incorporated with a semiquantitative food frequency questionnaire. Five single nucleotide polymorphisms (SNPs) of *GSTP1* (rs1695, rs749174, rs1871042, rs4891, and rs947895) were selected among the exome array genotype data.

**Results:**

High dietary ORAC was inversely associated with gastric cancer (hydrophilic ORAC OR _T3_ *_vs_*_. T1_, 95% CI = 0.57, 0.39–0.82, *P* = 0.004; lipophilic ORAC = 0.66, 0.45–0.95, *P* = 0.021; total phenolics = 0.57, 0.39–0.83, *P* = 0.005). The polymorphism rs1871042 increased the risk of gastric cancer (OR, 95% CI = 1.55, 1.10–2.16, *P* = 0.01, CT+TT *vs*. CC). A remarkably reduced risk of gastric cancer was observed among those who had a high dietary ORAC according to rs1871042 polymorphism (hydrophilic ORAC OR _T3_ *_vs_*_. T1_, 95% CI = 0.36, 0.17–0.78, *P* for trend = 0.013; lipophilic ORAC = 0.58, 0.37–0.93, *P* for trend = 0.021; total phenolics = 0.38, 0.17–0.83, *P* for trend = 0.019).

**Conclusions:**

Our findings indicate that dietary ORAC intake may be inversely associated with the risk of gastric cancer altered by genetic variants of *GSTP1*, providing new intervention strategies for gastric cancer patients.

## Background

Gastric cancer (GC) was the leading cause of cancer death and the fifth most common cancer worldwide in 2018 (1). Although the global incidence rates of GC have declined, the incidence of GC in East Asia, including Korea, remains high (1, 2). There are several major risk factors for the development of GC, including *Helicobacter pylori* (*H. pylori)* infection, smoking, alcohol consumption, obesity, and excess sodium intake (2). Generally, *H. pylori* infection is a known carcinogen and a strong risk factor for non-cardia GC by the classical histopathologic Correa cascade, consequently resulting in GC (3–5). *Helicobacter pylori* infection is particularly associated with an increased risk of not only non-cardia GC but also cardia GC according to several studies targeting East Asian countries, such as Korea (5, 6). In addition, evidence suggests that gastritis derived from chronic inflammation of normal mucosa may be linked to various other dietary factors, such as a high intake of salted or preserved foods and grilled or processed meats and a low intake of fruits (7, 8).

Dietary effects have been reported to mediate the risk of cancer by playing a role in either the prevention of cellular carcinoma or diet-induced carcinogenesis (9, 10). *Helicobacter pylori* infection is required to consider the causes of GC derived from either direct or indirect inflammation in the gastric mucosa (11, 12). Cumulative studies have reported that multiple *H. pylori*-induced inflammatory responses from reactive oxygen species (ROS) can be suppressed by bioactive compounds abundant in fruits and vegetables (13, 14). Moreover, current studies have consistently demonstrated that a variety of bioactive compounds, such as phytochemicals, play pivotal roles in sympathetic activation ranging from the inhibition of cellular proliferation to the suppression of metastasis in gastric carcinoma cells (15–17). Given that the risk of GC is linked to dietary factors and the inflammatory response associated with *H. pylori* infection, this study focused on exploring the integrated and synergistic effects of antioxidants on GC using the oxygen radical absorbance capacity (ORAC) of a diet. The ORAC is an experimental value representing the total antioxidant capacity (TAC) and is used to indicate the capacity to scavenge free radicals from food components indicating hydrophilic ORAC (H-ORAC), lipophilic ORAC (L-ORAC), and total phenolics (TPs) (18). The use of ORAC to assess dietary effects on disease has the advantage of exploring the antioxidant activity of food rather than that of a specific nutrient (19). Based on the benefits of using ORAC, an examination of the antioxidant capacity of a diet using this metric in the context of GC risk with inflammation and *H. pylori* infection is needed.

Among the inflammation- and oxidative stress-related genes, glutathione S-transferase Pi (GSTP1) is a cytosolic detoxifying enzyme that encodes Pi-class glutathione S-transferases (GST) and is involved in phase II xenobiotic metabolism by conjugating glutathione with hydrophobic and electrophilic substrates (20–22). The deregulation of *GSTP1* contributes to inducing oxidative stress by producing excessive ROS, leading to various types of tumors, including esophageal, stomach, lung, breast, and colorectal cancer (23–31). Regarding the risk of GC, studies exploring genetic polymorphisms of *GSTP1* revealed the role of *GSTP1* in and relevance of *GSTP1* for GC susceptibility (26, 27). Some epidemiological studies have reported a significant association between *GSTP1* rs1695 and the risk of GC (32, 33). One genetic polymorphism of *GSTP1*, namely, c.313 A > C (rs1695) in exon 5, results in an amino acid change (A to G) of isoleucine (Ile) to valine (Val), leading to impaired detoxification and catalytic activity (34). Furthermore, different genotypes of *GSTP1* rs1695 have significant interaction effects with environmental factors, including *H. pylori* infection, smoking, and alcohol consumption on the risk of GC (35–37). However, evidence regarding the associations between dietary factors and *GSTP1* polymorphisms in the context of GC risk based on the effects of antioxidants and imbalanced oxidative stress mechanisms is insufficient.

Given these points, we selected five single nucleotide polymorphisms (SNPs; rs1695, rs749174, rs1871042, rs4891, and rs947895) in *GSTP1* that were found among the 713,348 SNPs assayed in a Korean population based on quality control (QC) criteria in genome-wide association studies (GWASs), determined the association between ORAC and GC, and examined whether this association was modified by *GSTP1*. The aim of this study was to identify how dietary ORAC intake is associated with GC risk alterations by *GSTP1* genetic variants. We evaluated whether dietary ORAC intake affects the risk of GC. Additionally, we explored the associations between GC risk and ORAC intake according to *GSTP1* genotypes.

## Materials and Methods

### Study Population

**Figure 1:**
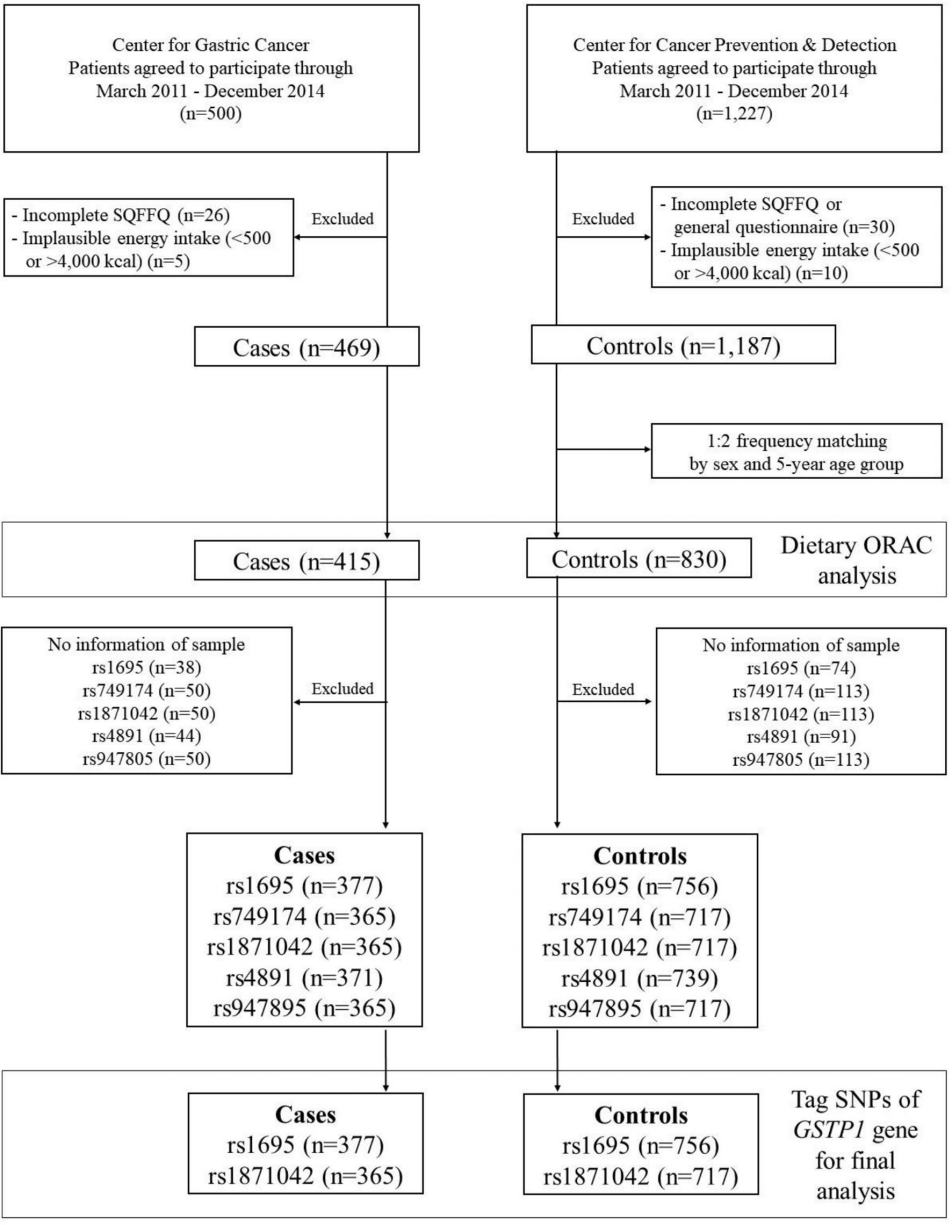
Flow chart of study subjects.

This case-control study was conducted at the Center for Gastric Cancer (CGC) and the Center for Cancer Prevention & Detection (CCPD) of the National Cancer Center (NCC) in Korea between March 2011 and December 2014. The cases were recruited among patients who were diagnosed with early GC within the preceding 3 months with confirmed invasive carcinoma in the CGC. Individuals with diabetes mellitus, severe systemic or mental disease, or a history of cancer and women who were pregnant or breastfeeding were excluded. The control group comprised individuals who underwent health screening check-ups at the CCPD at the same hospital. Participants in the control group who had diabetes mellitus, gastric or duodenal ulcers, a history of cancer, or previous *H. pylori* treatment were excluded. Of these initial 1,727 participants who were enrolled in this study, 56 individuals with an incomplete semiquantitative food frequency questionnaire (SQFFQ) and 15 individuals with an implausible total energy intake (< 500 or > 4,000 kcal/day) were excluded. Among the remaining 1,656 participants, the cases and controls were frequency-matched at a ratio of 1:2 (case: control) by 5-year age groups and sex. Regarding the genetic variants of *GSTP1*, we excluded low-quality samples and markers from the cases and controls as follows: rs1695 (n = 38 and n = 74), rs749174 (n = 50 and n = 113), rs1871042 (n = 50 and n = 113), rs4891 (n = 44 and n = 91), and rs947895 (n = 50 and n = 113). Consequently, the total population included the final analysis was as follows: rs1695 (n = 1,133), rs749174 (n = 1,082), rs1871042 (n = 1,082), rs4891 (n = 1,110), and rs947895 (n = 1,082) (**Figure 1**). This study was approved by the Institutional Review Board (IRB) of NCC (IRB number: NCCNCS-11-438), and written informed consent was obtained from all participants.

### Data Collection and Dietary Antioxidant Capacity Based on the ORAC Database

The sociodemographic characteristics were collected from each participant using a self-administered questionnaire. The status of *H. pylori* infection was assessed histologically or serologically from at least a positive result on a rapid urease test (Pronto Dry, Medical Instruments Corporation, Solothurn, Switzerland). The dietary intake data were obtained using a validated and reliable 106-item SQFFQ administered by a well-trained interviewer (38). The daily nutrient intake was calculated based on a combination of average intake frequency (never or rarely, 1 time per month, 2–3 times per month, 1–2 times per week, 3–4 times per week, 5–6 times per week, one time per day, two times per day, and three times per day) and the portion size (small, medium, and large) using CAN-PRO 4.0 (computer aided nutritional analysis program, Korean Nutrition Society, Seoul, Korea).

To estimate the values of the dietary antioxidant capacity, the ORAC database from the USDA release 2 was incorporated into our dietary intake data according to the food description (39). Our previous study reported the associations between dietary ORAC intake and interleukin-6 levels regarding the risk of colorectal cancer (40). Briefly, the ORAC database contains the antioxidant activity level of 326 food items. To calculate the dietary ORAC of each participant, the 106-item SQFFQ was integrated into the ORAC database by common food items. Of these food sources, 56 food items, including mostly fruits and vegetables, from the SQFFQ were considered to have antioxidant compounds and selected for the analysis in this study. It reported H-ORAC and L-ORAC as μmol of trolox equivalents per 100 g (μmol TE/100 g) and TPs as mg gallic acid equivalents per 100 g (mg GAE/100 g). We calculated each daily index of ORAC through the same process applied in the daily nutrient intake.

### Genotyping and SNP Selection

In this study, the SNPs of *GSTP1* were selected based on our previous study using exome array genotype data as described elsewhere (41). The genomic DNA samples were extracted from the peripheral blood leukocytes of all participants. The genotyping was performed using an Affymetrix Axiom^®^ Exome 319 Array containing 318,983 SNPs (Affymetrix Inc., Santa Clara, CA, USA). In the QC procedure applied to genotype data, the samples and genetic markers were excluded according to the call rate (< 95%), deviation from HWE, and MAF (< 0.01) (41, 42). After genotype imputation with an Asian population of 1000 Genome haplotypes phase III, we selected five SNPs of *GSTP1* (rs1695, rs749174, rs1871042, rs4891, and rs947895) for further analysis (**Supplementary Table S1**). The LD patterns of the SNPs were analyzed for the efficient selection of tag SNPs in *GSTP1* according to the pairwise D’ and r^2^ using Haploview (43).

### Statistical Analysis

The differences in the sociodemographic, anthropometric, lifestyle factors, and total energy intake between the cases and controls were assessed by using the χ^2^ test for categorical variables and Student’s *t*-test for continuous variables. The dietary ORAC intake and foods contributing to each ORAC, covering up to 90% of the cumulative contribution of 56 food items, were compared between the cases and controls by using a Wilcoxon signed-rank test because of its distribution (**Supplementary Tables S2**–**S4**). Energy-adjusted ORAC values and their contributing foods adjusted by a residual method were used in the analyses (44). The values of H-ORAC, L-ORAC, and TPs were divided into three groups depending on the median value of the controls. To analyze the associations among dietary ORAC intake, *GSTP1* polymorphisms and GC risk, unconditional logistic models were constructed to estimate the odds ratios (OR) and 95% confidence intervals (95% CI) of the risk of GC while considering potential confounding factors, such as age, BMI, education level, income, physical activity, smoking status, first-degree family history of GC, and total energy intake identified by the backward selection procedure in a stepwise regression analysis. The *H. pylori* infection status was additionally considered in the final statistical models. The analyses of the associations between dietary ORAC intake and GC risk were also stratified by *GSTP1* SNPs, particularly in the dominant model. All tests were performed using the SAS package (SAS 9.4; SAS Institute Inc., Cary, NC, USA) with a two-sided *P*-value of 0.05 regarded as significant.

## Results

### General Characteristics

**Table 1** shows the distribution of the sociodemographic characteristics and dietary ORAC intake in the cases and controls. The GC patients had a higher prevalence of positive *H. pylori* infection, a first-degree family history of GC and current smoking status, and a lower prevalence of regular exercise, education level, and income than the control group (*P* < 0.05). However, no differences in age, sex, BMI, or alcohol consumption were observed.

**Table 1:** General characteristics of study subjects.

	Controls (n=830)	Cases (n=415)	*P*-value^a^
Age (years)			
Mean ± SD	53.7 ± 9.0	53.8 ± 9.3	0.89
Sex (n, %)			
Male	540 (65.1)	270 (65.1)	>0.99
Female	290 (34.9)	145 (34.9)	
BMI (kg/m^2^) (n, %)			
<25	563 (67.9)	281 (67.9)	0.99
≥25	266 (32.1)	133 (32.1)	
*H. pylori* infection (n, %)			
Positive	486 (60.3)	382 (92.1)	<0.001
Negative	320 (39.7)	33 (8.0)	
Family history of GC (n, %) ^b^			
Yes	103 (12.4)	82 (19.8)	<0.001
No	725 (87.6)	332 (80.2)	
Physical activity (n, %)			
Yes	466 (56.4)	147 (35.4)	<0.001
No	361 (43.7)	268 (64.6)	
Smoking status (n, %)			
Current smoker	162 (19.5)	128 (30.9)	<0.001
Ex-smoker	284 (34.2)	119 (28.7)	
Non-smoker	384 (46.3)	167 (40.3)	
Alcohol consumption (n, %)			
Current drinker	534 (64.3)	254 (61.4)	0.24
Ex-drinker	60 (7.2)	41 (9.9)	
Non-drinker	236 (28.4)	119 (28.7)	
Education (n, %)			
Less than college	372 (46.6)	316 (76.5)	<0.001
College and higher	426 (53.4)	97 (23.5)	
Income (10,000 won/month) (n, %)			
<200	149 (19.5)	133 (35.3)	<0.001
200- < 400	341 (44.7)	148 (39.3)	
≥400	273 (35.8)	96 (25.5)	
			
Total energy (kcal/day)	1,713.59 ± 545.52	1,924.11 ± 612.91	<0.001
Dietary ORAC ^c^			
H-ORAC (μmol TE/day)	4,485.77 ± 3,371.96	3,443.90 ± 2,988.95	<0.001
Median (IQR)	3,598.63 (2,222.82, 5,666.39)	2,577.97 (1,637.04, 4,037.94)	
L-ORAC (μmol TE/day)	197.75 ± 117.55	166.75 ± 97.75	<0.001
Median (IQR)	176.08 (118.16, 244.92)	146.18 (100.28, 208.92)	
TPs (mg GAE/day)	423.17 ± 367.41	307.85 ± 282.93	<0.001
Median (IQR)	319.78 (192.53, 537.90)	216.70 (145.00, 368.61)	

ORAC, oxygen radical absorbance capacity; H-ORAC, hydrophilic oxygen radical absorbance capacity; L-ORAC, lipophilic oxygen radical absorbance capacity; TPs, total phenolics; TE, trolox equivalents; GAE, gallic acid equivalents; IQR, interquartile range. ^a^P-values were calculated the χ^2^ test for the categorical variables and a t-test for the continuous variables. ^b^ First-degree. ^c^Dietary ORAC were adjusted for the total energy intake using the residual method, and the p-values were calculated using a Wilcoxon signed-rank test.

The cases had a higher intake of daily total energy than the controls (1,924.11 ± 612.91 kcal/day *vs*. 1,713.59 ± 545.52 kcal/day, *P* < 0.001). Regarding the three components of dietary ORAC intake, the mean and median values of H-ORAC, L-ORAC, and TPs in the cases were lower than those in the controls (mean intake; H-ORAC, 3,443.90 ± 2,988.95 μmol TE/day *vs*. 4,485.77 ± 3,371.96 μmol TE/day, *P* < 0.001; L-ORAC, 166.75 ± 97.75 μmol TE/day *vs*. 197.75 ± 117.55 μmol TE/day, *P* < 0.001; TPs, 307.85 ± 282.93 mg GAE/day *vs*. 423.17 ± 367.41 mg GAE/day, *P* < 0.001, cases *vs*. controls).

### Association Between Dietary ORAC Intake and GC Risk

The associations between each index of dietary ORAC intake and GC risk are presented in **Table 2**. Compared to the lowest tertiles of H-ORAC, L-ORAC, and TPs, the highest tertiles of these three indices were significantly associated with GC risk after adjusting for all confounding factors. A decreased risk of GC was observed in those with a higher dietary ORAC intake as follows: H-ORAC (OR _T3_ *_vs_*_. T1_, 95% CI = 0.57, 0.39–0.82, *P* = 0.004); L-ORAC (OR _T3_ *_vs_*_. T1_, 95% CI = 0.66, 0.45–0.95, *P* = 0.021); and TPs (OR _T3_ *_vs_*_. T1_, 95% CI = 0.57, 0.39–0.83, *P* = 0.005).

**Table 2:** Association between dietary oxygen radical absorbance capacity (ORAC) intake and gastric cancer (GC) risk.

	Dietary ORAC			*P* for trend
	T1	T2	T3	
**H-ORAC (μmol TE/day)**	<2,655.07	2,655.07–4,759.10	≥4,759.11	
**No. Controls/Cases**	277/215	276/117	277/83	
**Model I OR (95% CI)**	1.0 (ref)	**0.55 (0.41–0.72)**	**0.39 (0.29–0.52)**	**<0.001**
**Model II OR (95% CI)**	1.0 (ref)	**0.61 (0.44–0.85)**	**0.54 (0.38–0.76)**	**<0.001**
**Model III OR (95% CI)**	1.0 (ref)	**0.65 (0.46–0.92)**	**0.57 (0.39–0.82)**	**0.004**
**L-ORAC (μmol TE/day)**	<139.97	139.97–215.48	≥215.49	
**No. controls/cases**	276/190	277/133	277/92	
**Model I OR (95% CI)**	1.0 (ref)	**0.70 (0.53–0.92)**	**0.48 (0.36–0.65)**	**<0.001**
**Model II OR (95% CI)**	1.0 (ref)	0.74 (0.54–1.02)	**0.53 (0.44–0.88)**	**0.007**
**Model III OR (95% CI)**	1.0 (ref)	0.72 (0.51–1.01)	**0.66 (0.45–0.95)**	**0.021**
**TPs (mg GAE/day)**	<230.09	230.09–445.51	≥445.52	
**No. controls/cases**	277/220	276/117	277/78	
**Model I OR (95% CI)**	1.0 (ref)	**0.53 (0.40–0.71)**	**0.36 (0.26–0.48)**	**<0.001**
**Model II OR (95% CI)**	1.0 (ref)	**0.61 (0.44–0.84)**	**0.52 (0.37–0.75)**	**0.001**
**Model III OR (95% CI)**	1.0 (ref)	**0.64 (0.45–0.90)**	**0.57 (0.39–0.83)**	**0.005**

ORAC, oxygen radical absorbance capacity; H-ORAC, hydrophilic oxygen radical absorbance capacity; L-ORAC, lipophilic oxygen radical absorbance capacity; TPs, total phenolics; TE, trolox equivalents; GAE, gallic acid equivalents; T, tertile; OR, odds ratio; 95% CI, 95% confidence interval. Model I: crude OR; model II: age (continuous), BMI (<25 kg/m^2^ or ≥25 kg/m^2^), education level (less than college or college and higher), income (<200, 200- <400 or ≥400), physical activity (yes or no), smoking status (current, ex- or non-smoker), first-degree family history of GC (yes or no), and total energy intake; model III: additionally, adjusted for H. pylori infection (positive or negative). Bold values mean the significant values (p<0.05) for the visual effect.

### Haplotype of *GSTP1* Polymorphisms and Association With GC Risk

**Figure 2:**
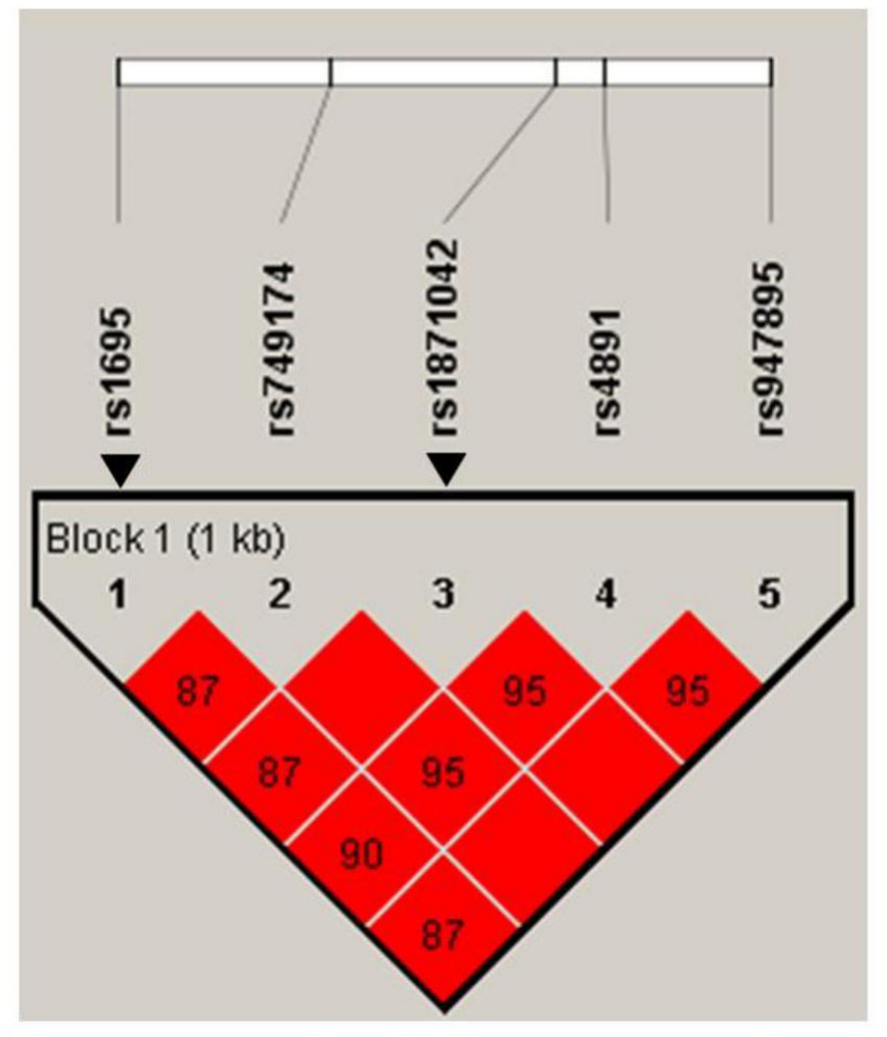
Haploview linkage disequilibrium (LD) patterns of *GSTP1* polymorphisms in chromosome 11. Pairwise LD is expressed as D’ (colors) and r2 (numbers). Arrows indicate Tag SNPs of *GSTP1* gene.

The minor allele frequencies of the five SNPs (rs1695, rs749174, rs1871042, rs4891, and rs947895) were common (minor allele frequency, MAF > 5%), and the genotype frequencies of the SNPs were consistent with Hardy-Weinberg equilibrium (HWE) (**Supplementary Table S1**). We identified the linkage disequilibrium (LD) structure of the five SNPs using a pairwise LD test (**Figure 2**). We observed that all five SNPs of *GSTP1* gene were in the same block with high LD, supporting the strong correlation among the five SNPs in this study. According to the LD patterns with r^2^ > 0.8, tag SNPs (rs1695 and rs1871042) in *GSTP1* were selected. **Table 3** presents the associations between *GSTP1* variants and GC risk. Apart from *H. pylori* infection, an increased risk of GC was found among those with heterozygous variants of *GSTP1*, namely, rs1871042 (OR, 95% CI = 1.60, 1.14–2.23, *P* = 0.006, CT *vs*. CC). When genetic models of each SNP were compared, the dominant model showed significant associations, but the recessive model did not. In the dominant model, an increased risk of GC was observed in those who carried T allele of rs1871042 (OR, 95% CI = 1.56, 1.13–2.16, *P* = 0.007, CT+TT *vs*. CC). After adjusting for confounders, a heterozygous variant of rs1871042 showed a significant association with GC risk (OR, 95% CI = 1.58, 1.12–2.25, *P* = 0.01, CT *vs*. CC). In the comparisons within the dominant genetic model, a modest and borderline association was observed between rs1871042 of *GSTP1* and GC risk (OR, 95% CI = 1.55, 1.10–2.16, *P* = 0.010, CT+TT *vs*. CC). However, there was no association between rs1695 polymorphism and GC risk. Additionally, the remaining SNPs in *GSTP1* showed associations with GC risk in the dominant genetic model as follows: rs749174 (OR, 95% CI = 1.55, 1.11–2.17, *P* = 0.010, GA+AA *vs*. GG); rs4891 (OR, 95% CI = 1.52, 1.09–2.10, *P* = 0.012, TC+CC *vs*. TT); and rs947895 (OR, 95% CI = 1.55, 1.10–2.16, *P* = 0.011, CA+AA *vs*. CC) (**Supplementary Table S5**).

**Table 3:** Association between tag single nucleotide polymorphisms (SNPs) of *GSTP1* gene and gastric cancer (GC) risk.

*GSTP1* SNPs	No. controls/cases	Model I OR (95% CI)	*P*-value^a^	Model II OR (95% CI)	*P*-value^a^	Model III OR (95% CI)	*P*-value^a^
**rs1695**							
**AA**	505/242	1.0 (ref)		1.0 (ref)		1.0 (ref)	
**AG**	225/116	1.08 (0.82–1.41)	0.60	1.34 (0.98–1.84)	0.07	1.33 (0.95–1.85)	0.08
**GG**	26/19	1.53 (0.83–2.81)	0.18	1.42 (0.69–2.90)	0.34	1.44 (0.68–3.08)	0.34
**Dominant**							
**AA**	505/242	1.0 (ref)		1.0 (ref)		1.0 (ref)	
**AG+GG**	251/135	1.12 (0.87–1.46)	0.38	1.35 (1.00–1.83)	0.05	1.34 (0.98–1.85)	0.07
**Recessive**							
**AA+AG**	730/358	1.0 (ref)		1.0 (ref)		1.0 (ref)	
**GG**	26/19	1.49 (0.81–2.73)	0.20	1.29 (0.63–2.63)	0.48	1.32 (0.62–2.79)	0.47
**rs1871042**							
**CC**	523/247	1.0 (ref)		1.0 (ref)		1.0 (ref)	
**CT**	174/107	1.30 (0.98–1.73)	0.07	**1.60 (1.14–2.23)**	**0.006**	**1.58 (1.12–2.25)**	**0.01**
**TT**	20/11	1.17 (0.55–2.47)	0.69	1.31 (0.55–3.08)	0.54	1.25 (0.51–3.06)	0.63
**Dominant**							
**CC**	523/247	1.0 (ref)		1.0 (ref)		1.0 (ref)	
**CT+TT**	194/118	1.29 (0.98–1.69)	0.07	**1.56 (1.13–2.16)**	**0.007**	**1.55 (1.10–2.16)**	**0.010**
**Recessive**							
**CC+CT**	697/354	1.0 (ref)		1.0 (ref)		1.0 (ref)	
**TT**	20/11	1.08 (0.51–2.29)	0.83	1.15 (0.49–2.70)	0.75	1.10 (0.45–2.67)	0.84

OR, odds ratio; 95% CI, 95% confidence interval. ^a^P-values were calculated using the χ^2^ test. Model I: crude OR; model II: age (continuous), BMI (<25 kg/m^2^ or ≥25 kg/m^2^), education level (less than college or college and higher), income (<200, 200- <400 or ≥400), physical activity (yes or no), smoking status (current, ex- or non-smoker), first-degree family history of GC (yes or no), and total energy intake; model III: additionally, adjusted for H. pylori infection (positive or negative). Bold values mean the significant values (p<0.05) for the visual effect.

### Association Between Dietary ORAC Intake and GC Risk by rs1871042 Polymorphism of *GSTP1* Gene

**Table 4** shows the associations between dietary intake and GC risk in the dominant model of rs1871042 *GSTP1* polymorphism. Inverse associations between dietary H-ORAC intake and GC risk were observed among those with the rs1871042 T allele after fully adjusting for potential confounders (OR _T3_ *_vs_*_. T1_, 95% CI = 0.36, 0.17–0.78, *P* = 0.013). The analysis showed a similar pattern between dietary TPs intake and GC risk (OR _T3_ *_vs_*_. T1_, 95% CI = 0.38, 0.17–0.83, *P* = 0.019). However, a high intake of dietary L-ORAC decreased the GC risk with a homozygous variant of CC genotype (OR _T3_ *_vs_*_. T1_, 95% CI = 0.58, 0.37–0.93, *P* = 0.021). Without considering *H. pylori* infection status, higher H-ORAC and TPs intake were consistently associated with a decreased risk of GC with rs1871042 T allele (H-ORAC OR _T3_ *_vs_*_. T1_, 95% CI = 0.31, 0.15–0.64, *P* = 0.003; TPs OR _T3_ *_vs_*_. T1_, 95% CI = 0.34, 0.16–0.71, *P* = 0.005). Moreover, higher L-ORAC and TPs intake was associated with a reduced risk of GC in those with the minor allele (L-ORAC OR _T3_ *_vs_*_. T1_, 95% CI = 0.58, 0.37–0.91, *P* = 0.016; TPs OR _T3_ *_vs_*_. T1_, 95% CI = 0.61, 0.38–0.97, *P* = 0.046). The remaining SNPs (rs749174, rs4891, and rs947895) in *GSTP1* showed similar patterns of association with dietary ORAC intake on GC risk (**Supplementary Table S6**). Regarding the interaction effect between dietary ORAC intake and *GSTP1* rs1871042 polymorphism on gastric cancer risk, each dietary ORAC intake was divided into low and high groups based on the median level of the intake of controls (**Supplementary Table S7**). Although there are no interaction effects between dietary ORAC intake and *GSTP1* rs1871042 polymorphism on gastric cancer, a high intake of ORAC significantly reduced the risk of gastric cancer in patients homozygous for CC at rs1871042 after adjusting for potential confounding factors (OR, 95% CI: H-ORAC = 0.63, 0.44–0.91; L-ORAC = 0.60, 0.42–0.87; TPs = 0.68, 0.47-0.99). However, a low intake of H-ORAC and TPs while carrying a T allele (CT+TT) increased the risk of gastric cancer compared with that observed in the CC homozygous patients (OR, 95% CI: H-ORAC = 1.64, 1.08–2.49; TPs = 1.67, 1.11–2.53).

**Table 4:** Association between dietary oxygen radical absorbance capacity (ORAC) intake and gastric cancer (GC) risk by rs1871042 polymorphism of *GSTP1* gene.

GSTP1		No. controls/cases		Model I OR (95% CI)		Model II OR (95% CI)		Model III OR (95% CI)	
rs1871042 (dominant)		CC	CT+TT	CC	CT+TT	CC	CT+TT	CC	CT+TT
H-ORAC (μmol TE/day)	T1 (<2,655.88)	175/122	61/65	1.0 (ref)	1.0 (ref)	1.0 (ref)	1.0 (ref)	1.0 (ref)	1.0 (ref)
	T2 (2,655.88–4,759.10)	175/73	64/31	0.60 (0.42–0.86)	0.46 (0.26–0.79)	0.75 (0.49–1.15)	**0.33 (0.16–0.68)**	0.77 (0.49-1.20)	**0.39 (0.19-0.82)**
	T3 (>4,759.10)	173/52	69/22	0.43 (0.29–0.64)	0.30 (0.17–0.54)	0.63 (0.40–1.00)	**0.31 (0.15–0.64)**	0.65 (0.41-1.05)	**0.36 (0.17-0.78)**
	*P* for trend			< 0.001	< 0.001	0.06	**0.003**	0.084	**0.013**
L-ORAC (μmol TE/day)	T1 (<140.01)	170/115	65/49	1.0 (ref)	1.0 (ref)	1.0 (ref)	1.0 (ref)	1.0 (ref)	1.0 (ref)
	T2 (140.01-215.68)	175/76	66/44	0.64 (0.45–0.92)	0.88 (0.52–1.51)	0.68 (0.44–1.03)	0.80 (0.41–1.53)	0.67 (0.43-1.03)	0.66 (0.33-1.33)
	T3 (>215.68)	178/56	63/25	0.47 (0.32–0.68)	0.53 (0.29–0.95)	**0.58 (0.37-0.91)**	0.80 (0.39–1.64)	**0.58 (0.37-0.93)**	0.86 (0.40-1.86)
	*P* for trend			< 0.001	0.035	**0.016**	0.524	**0.021**	0.642
TPs (mg GAE/day)	T1 (<230.58)	170/127	67/65	1.0 (ref)	1.0 (ref)	1.0 (ref)	1.0 (ref)	1.0 (ref)	1.0 (ref)
	T2 (230.58-445.51)	175/69	62/33	0.53 (0.37–0.76)	0.55 (0.32–0.94)	0.66 (0.43–1.01)	**0.47 (0.24–0.94)**	0.70 (0.45-1.09)	**0.45 (0.22-0.92)**
	T3 (>445.51)	178/51	65/20	0.38 (0.26–0.57)	0.32 (0.17–0.58)	**0.61 (0.38–0.97)**	**0.34 (0.16–0.71)**	0.64 (0.40-1.04)	**0.38 (0.17-0.83)**
	*P* for trend			< 0.001	< 0.001	**0.046**	**0.005**	0.087	**0.019**

ORAC, oxygen radical absorbance capacity; H-ORAC, hydrophilic oxygen radical absorbance capacity; L-ORAC, lipophilic oxygen radical absorbance capacity; TPs, total phenolics; TE, trolox equivalents; GAE, gallic acid equivalents; T, tertile; OR, odds ratio; 95% CI, 95% confidence interval. Model I: crude OR; model II: age (continuous), BMI (<25 kg/m^2^ or ≥25 kg/m^2^), education level (less than college or college and higher), income (<200, 200- <400 or ≥400), physical activity (yes or no), smoking status (current, ex- or non-smoker), first-degree family history of GC (yes or no), and total energy intake; Model III: additionally adjusted for H. pylori infection (positive or negative).

## Discussion

The present study aimed to determine the association between dietary ORAC intake and GC risk according to *GSTP1* genetic variants. A high dietary intake of ORAC was significantly associated with a decreased risk of GC in a Korean population. Regarding the genetic variants of *GSTP1* gene, dietary ORAC intake was inversely associated with GC risk according to *GSTP1* rs1871042 genotypes.

Gastric adenocarcinoma occurs when normal mucosa cells are continuously exposed to a variety of carcinogens that lead to uncontrolled cell proliferation in the gastric mucosa membrane (45). The following two major mechanisms are linked to the development of GC with *H. pylori* infection: (1) epigenetic alterations in gastric epithelial cells by *H. pylori* infection and (2) *H. pylori*-induced inflammation in the gastric mucosa (46). Many studies have shown that persistent inflammation, through cytokines, chemokines, growth factors, and oxygen-derived free radicals is responsible for GC risk associated with *H. pylori* infection (46, 47). The role of oxidative stress from inflammation in GC has been determined, suggesting the importance of a balance between radical production and the antioxidant defense system (48). Numerous studies have reported that the intake of fruits and vegetables is inversely associated with GC risk, while some studies found no such associations (49–53). Specifically, a high intake of fruits by *H. pylori*-negative subjects decreased the risk of GC compared to a low intake of fruits by *H. pylori*-positive subjects, indicating that the intake of fruits and vegetables may play a role in preventing *H. pylori*-induced gastric carcinogenesis (52–54). In contrast, data regarding the effects of vitamin A, vitamin C, vitamin E, and carotenoids on GC risk were inconsistent or conflicting due to the different doses used (55–57). In this study, we examined the antioxidant capacity of food and determined the antioxidant effects of ORAC on gastric carcinogenesis. A recent meta-analysis reported inverse associations between cancer risk and dietary TAC by using multiple methods, including ORAC (58). Other previous studies found inverse associations between ORAC intake and risk of other cancers but not GC (59–62). We observed similar findings between GC risk and three indices of dietary ORAC, namely, H-ORAC, L-ORAC, and TPs, after adjusting for *H. pylori* infection and other potential confounding factors. Furthermore, in the comparisons of the food items that highly contribute to the ORAC level, the food items with the highest ORAC were brewed green tea and fruits for H-ORAC, spicy red, or black pepper for L-ORAC, and canned tomato juice for TPs (**Supplementary Tables S2**–**S4**).

The major function of GSTP1 is to detoxify exogenous or endogenous factors involved in carcinogenesis by regulating cell death and DNA damage (21, 63). Additionally, GSTP1 plays a role as a modifier gene in the regulation of the molecular expression and activation of enzymes from other GST subfamilies and their effects on cancer, and GSTP1 expression regulates cellular redox homeostasis in carcinogenesis (20, 64, 65). Although many studies have shown associations between *GSTP1* polymorphisms and various types of cancer, the results of a few studies investigating the associations between GC risk and *GSTP1* genetic variants are inconsistent across geographic areas and diverse populations. In a Chinese population, the Val allele of *GSTP1*, namely, the Val/Val genotype, was significantly associated with an increased risk of GC (37, 66, 67). However, *GSTP1* Ile105Val (rs1695) and *GSTP1* Val114Ala (rs1138272) polymorphisms were not associated with the risk of GC in either a South European or an Indian population (68, 69). In a Korean population, we observed that five *GSTP1* polymorphisms (rs1695, rs749174, rs1871042, rs4891, and rs947895) located in the same block with a strong correlation with high LD had a tendency to increase GC risk, although the risk increase with rs1695 polymorphism was not statistically significant. These conflicting results suggest that ethnic differences in *GSTP1* genetic susceptibility may affect the development of GC with epigenetic interactions of environmental factors and that the relevance of *GSTP1* genetic variants to GC risk needs to be confirmed in future studies. Among five *GSTP1* polymorphisms examined in this study, four polymorphisms (rs749174, rs1871042, rs4891, and rs947895) have been investigated in only a few studies in the context of lung cancer and asthma, and to date, their associations with GC risk have not been determined (70–72).

In this study, we observed an association between a high intake of dietary ORAC and a reduced GC risk according to *GSTP1* rs1871042 polymorphism. Our findings can be explained by the interconnections between dietary TAC and the role of *GSTP1* gene in the regulation of oxidative stress and detoxification of the immune response against gastric carcinogenesis-induced chronic inflammation by *H. pylori* infection. Imbalanced oxidative stress plays an obligatory role in gastric carcinogenesis by increasing the level of ROS induced by *H. pylori* infection, leading to DNA damage and tumor progression (4, 73). A high intake of dietary ORAC is responsible for the scavenging substances produced by *H. pylori*-infected gastric cells and, thus, may protect against the promotion of gastric carcinogenesis. More than half of *H. pylori* strains produce various cytotoxins, such as Cag-A, which can damage gastric mucosal cell membranes and trigger local immune responses (74). Previous studies have shown that vitamin C protects against *H. pylori* infection-related GC by neutralizing free radicals and directly modifying the anticancer immune response against malignant progression (75, 76). In addition to the role of *GSTP1* gene, the specific allele of *GSTP1* is able to regulate oxidative stress and detoxification against carcinogenesis (21, 65). Moreover, the impact of *H. pylori* infection on the relationship between *GSTP1* genetic polymorphisms and GC risk varies, suggesting that *H. pylori* infection may have different oncogenic effects depending on *GSTP1* genetic polymorphism, including controlling the activation of the detoxification system, thereby resulting in gastric carcinogenesis (36, 77). A high intake of dietary ORAC may synergistically interact with *GSTP1* rs1871042 polymorphism by detoxifying and eradicating excessive ROS, eventually leading to protection against the development of GC.

Nevertheless, some limitations should be noted. First, selection and recall bias should be considered; the controls were recruited among patients who visited the clinic for a health check-up and may have been more health conscious than the patients with GC. To reduce the selection bias, controls who were confirmed to be cancer-free by linking to the Korea Central Cancer Registry database were recruited. However, it may be that the individuals who chose to visit a health check-up program may have a healthier lifestyle than those who did not choose to undergo a check-up. Moreover, the participants provided the structured questionnaire and validated SQFFQ by a well-trained interviewer to reduce the recall bias. The SQFFQ includes the average intake frequency and the portion size during the year preceding the interview. Second, the food items included in our food database were insufficient to cover the entire United States Department of Agriculture (USDA) ORAC database. Additionally, the antioxidant capacity from ORAC is based on *in vitro* antioxidant assays, which are limited to measuring the absorption rate in the body. Third, the sample size in each tertile of the case group is relatively small. Further prospective studies are needed to confirm and extend our findings with a larger sample size.

## Conclusions

In conclusion, this study examined whether the associations between dietary ORAC and GC risk were modified by *GSTP1* polymorphisms. We found associations between the risk of GC and dietary ORAC intake, including fruits, vegetables, spices, and nuts, depending on the genetic variants of *GSTP1*. Considering the highest incidence rates of GC with *H. pylori* infection in East Asia, the associations among dietary ORAC intake, *GSTP1* polymorphisms, and GC risk may provide an effective strategy for the primary prevention of GC in Asian populations.

## Data Availability Statement

The datasets presented in this study can be found in online repositories. The names of the repository/repositories and accession number(s) can be found in the article/**Supplementary Material**.

## Ethics Statement

The studies involving human participants were reviewed and approved by the Institutional Review Board (IRB) NCC (IRB number: NCCNCS-11-438), and written informed consent was obtained from all participants. The patients/participants provided their written informed consent to participate in this study.

## Author Contributions

We greatly appreciate the efforts of those who contributed to this study. The authors’ responsibilities were as follows: JMK, HK, and JSK: designed the research. JMK, HK, JL, IC, Y-IK, and JSK: conducted the research. JMK and HK: analyzed the data. JMK, HK, and JSK wrote the paper and are primarily responsible for the final content. All authors contributed to the article and approved the submitted version.

## Funding

This work was supported by grants from the National Cancer Center (No. 1410260, 1810980, and 1910330) and National Research Foundation funded by the South Korean Government (2018R1D1A1A09083876).

## Conflict of Interest

The authors declare that the research was conducted in the absence of any commercial or financial relationships that could be construed as a potential conflict of interest.

## Abbreviations

BMI, body mass index; CCPD, Center for Cancer Prevention and Detection; CGC, Center for Gastric Cancer; CI, confidence intervals; GC, gastric cancer; GST, glutathione S-transferases; GSTP1, glutathione S-transferase Pi; GWASs, genome-wide association studies; H-ORAC, hydrophilic oxygen radical absorbance capacity; *H. pylori*, *Helicobacter pylori*; HWE, Hardy-Weinberg equilibrium; LD, linkage disequilibrium; L-ORAC, lipophilic oxygen radical absorbance capacity; MAF, minor allele frequency; NCC, National Cancer Center; OR, odds ratios; ORAC, oxygen radical absorbance capacity; QC, quality control; ROS, reactive oxygen species; SNP, single nucleotide polymorphism; SQFFQ, semiquantitative food frequency questionnaire; TAC, total antioxidant capacity; TPs, total phenolics.
