# Supplementary material for: Antioxidant-Rich Diet, GSTP1 rs1871042 Polymorphism, and Gastric Cancer Risk in a Hospital-Based Case-Control Study
Source: Front Oncol. 2021 Jan 29;10:596355. doi: 10.3389/fonc.2020.596355 (PMC7902036; doi:10.3389/fonc.2020.596355)
Supplement: Supplementary file 1 [file DataSheet_1.docx]

**Table S1. Description of *GSTP1* SNPs ^a^**

| **rsID** | **Chromosome**  **location** | **Allele**  **(Major/Minor)** | **Most severe consequence** | **MAF ^b^** | **HWE**  **p-value ^c^** |
| --- | --- | --- | --- | --- | --- |
| rs1695 | 11:67352688 | A/G | Missense variant | 0.35 (G) | 0.88 |
| rs749174 | 11:67353252 | G/A | Intron variant | 0.24 (A) | 0.24 |
| rs1871042 | 11:67353843 | C/T | Non coding transcript exon variant | 0.25 (T) | 0.24 |
| rs4891 | 11:67353969 | T/C | Synonymous variant | 0.36 (C) | 0.78 |
| rs947895 | 11:67354405 | C/A | Intergenic variant | 0.25 (A) | 0.24 |

MAF: Minor allele frequency, HWE: Hardy-Weinberg equilibrium. ^a^ Data on the NCBI dbSNP database (<http://www.ncbi.nlm.nih.gov/projects/SNP>). ^b^ Minor allele frequency in the controls of this study. ^c^ Hardy-Weinberg equilibrium *p*-values in the controls of this study

**Table S2. Food items contributing to H-ORAC between the control and case groups ^a^**

| **No.** | **Food (μmol TE/d)** | **Percentage (%)** | **Cumulative percentage (%)** | **Controls** | | | **Cases** | | | ***P*-value ^b^** |
| --- | --- | --- | --- | --- | --- | --- | --- | --- | --- | --- |
| 1 | Tea, green, brewed | 22.90 | 22.90 | 1658.18 | ± | 8310.27 | 891.23 | ± | 5756.16 | < 0.001 |
| 2 | Apples, Fuji, raw, with skin | 9.97 | 32.87 | 715.44 | ± | 1271.80 | 401.01 | ± | 756.85 | < 0.001 |
| 3 | Apples, raw, with skin | 7.89 | 40.76 | 566.31 | ± | 1004.76 | 317.96 | ± | 599.76 | < 0.001 |
| 4 | Radishes, raw | 5.89 | 46.65 | 366.21 | ± | 315.33 | 349.19 | ± | 301.54 | 0.717 |
| 5 | Strawberries, raw | 5.65 | 52.30 | 404.57 | ± | 790.71 | 229.25 | ± | 392.60 | < 0.001 |
| 6 | Apples, Red Delicious, raw, with skin | 4.90 | 57.20 | 351.32 | ± | 620.93 | 197.94 | ± | 372.97 | < 0.001 |
| 7 | Potatoes, white, flesh and skin, raw | 4.70 | 61.90 | 293.79 | ± | 299.57 | 275.53 | ± | 288.48 | 0.276 |
| 8 | Grapes, black | 4.22 | 66.12 | 270.22 | ± | 724.20 | 234.52 | ± | 1044.85 | < 0.001 |
| 9 | Bananas, raw | 2.18 | 68.30 | 161.14 | ± | 343.99 | 79.04 | ± | 200.08 | < 0.001 |
| 10 | Onions, raw | 2.17 | 70.47 | 134.58 | ± | 82.85 | 130.24 | ± | 78.59 | 0.560 |
| 11 | Lettuce, green leaf, raw | 2.08 | 72.55 | 139.07 | ± | 247.29 | 103.50 | ± | 151.76 | 0.001 |
| 12 | Soybeans, mature seeds, sprouted, raw | 1.94 | 74.49 | 124.16 | ± | 181.85 | 107.37 | ± | 127.66 | 0.022 |
| 13 | Tomato juice, canned | 1.78 | 76.26 | 134.48 | ± | 391.08 | 57.27 | ± | 125.38 | < 0.001 |
| 14 | Plums, raw | 1.76 | 78.02 | 128.58 | ± | 388.03 | 65.70 | ± | 163.57 | < 0.001 |
| 15 | Peanuts, all types, raw | 1.75 | 79.77 | 133.08 | ± | 496.88 | 55.59 | ± | 219.50 | < 0.001 |
| 16 | Lemon juice, raw | 1.71 | 81.48 | 106.81 | ± | 972.53 | 100.14 | ± | 1084.71 | < 0.001 |
| 17 | Orange juice, raw | 1.69 | 83.17 | 130.15 | ± | 391.35 | 50.74 | ± | 128.67 | 0.026 |
| 18 | Gallic, raw | 1.57 | 84.74 | 97.88 | ± | 68.66 | 92.45 | ± | 57.29 | < 0.001 |
| 19 | Spinach, raw | 1.41 | 86.15 | 68.57 | ± | 727.77 | 121.71 | ± | 1820.51 | 0.374 |
| 20 | Spices, pepper, red or cayenne | 1.02 | 87.17 | 65.98 | ± | 68.50 | 55.69 | ± | 51.77 | 0.003 |
| 21 | Cabbage, raw | 1.00 | 88.17 | 74.87 | ± | 164.28 | 34.65 | ± | 80.21 | 0.017 |
| 22 | Carrots, boiled | 0.98 | 89.15 | 69.96 | ± | 668.28 | 40.00 | ± | 544.56 | < 0.001 |
| 23 | Oranges, raw, all commercial varieties | 0.80 | 89.95 | 61.10 | ± | 180.00 | 24.23 | ± | 61.08 | 0.329 |
| 24 | Ginger root, raw | 0.67 | 90.62 | 51.07 | ± | 562.83 | 21.25 | ± | 311.28 | < 0.001 |

H-ORAC: hydrophilic oxygen radical absorbance capacity, TE: Trolox equivalents. ^a^ Each food component was adjusted for the total energy intake using the residual method. ^b^ *P*-values were calculated using a Wilcoxon signed-rank test.

**Table S3. Food items contributing to L-ORAC between the control and case groups ^a^**

| **No.** | **Food (μmol TE/d)** | **Percentage (%)** | **Cumulative percentage (%)** | **Controls** | | | **Cases** | | | ***P*-value ^b^** |
| --- | --- | --- | --- | --- | --- | --- | --- | --- | --- | --- |
| 1 | Spices, pepper, red or cayenne | 40.98 | 40.98 | 88.60 | ± | 92.06 | 74.74 | ± | 69.52 | 0.017 |
| 2 | Spices, pepper, black | 8.53 | 49.51 | 18.59 | ± | 23.15 | 15.26 | ± | 15.50 | 0.036 |
| 3 | Potatoes, white, flesh and skin, raw | 6.77 | 56.28 | 14.14 | ± | 14.26 | 13.35 | ± | 13.95 | 0.317 |
| 4 | Lettuce, green leaf, raw | 6.10 | 62.38 | 13.60 | ± | 23.43 | 10.30 | ± | 14.76 | 0.001 |
| 5 | Bananas, raw | 5.65 | 68.03 | 13.86 | ± | 28.66 | 7.00 | ± | 17.68 | < 0.001 |
| 6 | Peanuts, all types, raw | 4.30 | 72.33 | 10.86 | ± | 37.31 | 4.73 | ± | 16.75 | < 0.001 |
| 7 | Gallic, raw | 3.38 | 75.72 | 7.07 | ± | 4.96 | 6.67 | ± | 4.14 | 0.374 |
| 8 | Radishes, raw | 2.65 | 78.37 | 5.52 | ± | 4.75 | 5.27 | ± | 4.54 | 0.732 |
| 9 | Apples, raw, with skin | 2.41 | 80.77 | 5.73 | ± | 9.83 | 3.33 | ± | 6.25 | < 0.001 |
| 10 | Apples, Fuji, raw, with skin | 2.34 | 83.11 | 5.56 | ± | 9.55 | 3.23 | ± | 6.07 | < 0.001 |
| 11 | Mushroom, oyster, raw | 1.93 | 85.04 | 4.31 | ± | 9.59 | 3.24 | ± | 8.77 | < 0.001 |
| 12 | Watermelon, raw | 1.73 | 86.77 | 4.03 | ± | 13.42 | 2.56 | ± | 4.16 | < 0.001 |
| 13 | Apples, Red Delicious, raw, with skin | 1.36 | 88.13 | 3.24 | ± | 5.55 | 1.89 | ± | 3.55 | < 0.001 |
| 14 | Strawberries, raw | 1.35 | 89.48 | 3.21 | ± | 5.87 | 1.89 | ± | 3.15 | < 0.001 |
| 15 | Lettuce, cos or romaine, raw | 1.13 | 90.61 | 2.81 | ± | 5.75 | 1.36 | ± | 2.92 | < 0.001 |

L-ORAC: lipophilic oxygen radical absorbance capacity, TE: Trolox equivalents. ^a^ Each food component was adjusted for the total energy intake using the residual method. ^b^ *P*-values were calculated using a Wilcoxon signed-rank test.

.

**Table S4. Food items contributing to TPs between the control and case groups ^a^**

| **No.** | **Food (mg GAE/d)** | **Percentage (%)** | **Cumulative percentage (%)** | **Controls** | | | **Cases** | | | ***P*-value ^b^** |
| --- | --- | --- | --- | --- | --- | --- | --- | --- | --- | --- |
| 1 | Tomato juice, canned | 15.16 | 15.16 | 88.70 | ± | 256.62 | 38.01 | ± | 83.13 | < 0.001 |
| 2 | Apples, Fuji, raw, with skin | 10.30 | 25.46 | 56.93 | ± | 99.24 | 32.51 | ± | 61.08 | < 0.001 |
| 3 | Potatoes, white, flesh and skin, raw | 9.77 | 35.23 | 47.19 | ± | 47.79 | 44.44 | ± | 46.46 | 0.299 |
| 4 | Apples, raw, with skin | 8.27 | 43.49 | 45.69 | ± | 79.52 | 26.13 | ± | 49.09 | < 0.001 |
| 5 | Bananas, raw | 5.82 | 49.32 | 33.11 | ± | 69.23 | 16.55 | ± | 41.83 | < 0.001 |
| 6 | Strawberries, raw | 5.52 | 54.84 | 30.41 | ± | 57.33 | 17.59 | ± | 29.64 | < 0.001 |
| 7 | Apples, Red Delicious, raw, with skin | 5.08 | 59.91 | 28.04 | ± | 48.63 | 16.09 | ± | 30.22 | < 0.001 |
| 8 | Cabbage, raw | 4.99 | 64.91 | 28.74 | ± | 61.76 | 13.47 | ± | 30.45 | < 0.001 |
| 9 | Radishes, raw | 3.49 | 68.39 | 16.77 | ± | 14.43 | 16.00 | ± | 13.81 | 0.729 |
| 10 | Peanuts, all types, raw | 2.76 | 71.15 | 16.11 | ± | 56.07 | 6.96 | ± | 25.08 | < 0.001 |
| 11 | Catsup | 2.55 | 73.70 | 14.83 | ± | 41.95 | 6.53 | ± | 14.23 | < 0.001 |
| 12 | Carrots, boiled | 2.41 | 76.11 | 13.38 | ± | 120.28 | 7.51 | ± | 95.71 | 0.342 |
| 13 | Watermelon, raw | 2.35 | 78.46 | 12.71 | ± | 43.00 | 8.00 | ± | 13.13 | < 0.001 |
| 14 | Lemon juice, raw | 2.15 | 80.61 | 10.45 | ± | 89.85 | 9.63 | ± | 96.18 | 0.028 |
| 15 | Spices, pepper, red or cayenne | 1.77 | 82.38 | 8.83 | ± | 9.12 | 7.48 | ± | 6.93 | 0.021 |
| 16 | Lettuce, green leaf, raw | 1.67 | 84.05 | 8.62 | ± | 14.77 | 6.55 | ± | 9.35 | 0.001 |
| 17 | Spinach, raw | 1.53 | 85.58 | 6.19 | ± | 59.66 | 9.32 | ± | 132.21 | 0.003 |
| 18 | Soybeans, mature seeds, sprouted, raw | 1.22 | 86.80 | 6.05 | ± | 8.86 | 5.24 | ± | 6.21 | 0.025 |
| 19 | Plums, raw | 1.09 | 87.89 | 6.11 | ± | 16.59 | 3.32 | ± | 7.76 | < 0.001 |
| 20 | Raisins, seedless | 0.98 | 88.87 | 4.87 | ± | 11.47 | 4.23 | ± | 16.38 | < 0.001 |
| 21 | Squash, summer, zucchini, includes skin, raw | 0.95 | 89.82 | 4.56 | ± | 6.38 | 4.32 | ± | 5.91 | 0.338 |
| 22 | Melons, cantaloupe, raw | 0.87 | 90.69 | 4.90 | ± | 13.38 | 2.56 | ± | 4.88 | < 0.001 |

TPs: total phenolics, GAE: gallic acid equivalents. ^a^ Each food component was adjusted for the total energy intake using the residual method. ^b^ *P*-values were calculated using a Wilcoxon signed-rank test.

**Table S5. Association between *GSTP1* SNPs and GC risk**

| ***GSTP1***  **SNPs** | **No. Controls/Cases** | **Model I**  **OR (95% CI)** | ***P*-value ^a^** | **Model II**  **OR (95% CI)** | ***P*-value ^a^** | **Model III**  **OR (95% CI)** | ***P*-value ^a^** |
| --- | --- | --- | --- | --- | --- | --- | --- |
| **rs749174** |  |  |  |  |  |  |  |
| **GG** | 523/247 | 1.0 (ref) |  | 1.0 (ref) |  | 1.0 (ref) |  |
| **GA** | 174/107 | 1.30 (0.98-1.73) | 0.07 | 1.60 (1.15-2.24) | 0.006 | 1.59 (1.12-2.26) | 0.009 |
| **AA** | 20/11 | 1.17 (0.55-2.47) | 0.69 | 1.31 (0.55-3.09) | 0.54 | 1.25 (0.51-3.06) | 0.62 |
| **Dominant** |  |  |  |  |  |  |  |
| **GG** | 523/247 | 1.0 (ref) |  | 1.0 (ref) |  | 1.0 (ref) |  |
| **GA+AA** | 194/118 | 1.29 (0.98-1.69) | 0.07 | 1.57 (1.14-2.16) | 0.006 | 1.55 (1.11-2.17) | 0.010 |
| **Recessive** |  |  |  |  |  |  |  |
| **GG+GA** | 697/354 | 1.0 (ref) |  | 1.0 (ref) |  | 1.0 (ref) |  |
| **AA** | 20/11 | 1.08 (0.51-2.29) | 0.83 | 1.15 (0.49-2.70) | 0.75 | 1.10 (0.45-2.67) | 0.83 |
| **rs4891** |  |  |  |  |  |  |  |
| **TT** | 517/241 | 1.0 (ref) |  | 1.0 (ref) |  | 1.0 (ref) |  |
| **TC** | 201/116 | 1.24 (0.94-1.63) | 0.13 | 1.55 (1.12-2.15) | 0.008 | 1.53 (1.09-2.14) | 0.01 |
| **CC** | 21/14 | 1.43 (0.72-2.86) | 0.31 | 1.54 (0.68-3.48) | 0.30 | 1.45 (0.62-3.38) | 0.39 |
| **Dominant** |  |  |  |  |  |  |  |
| **TT** | 517/241 | 1.0 (ref) |  | 1.0 (ref) |  | 1.0 (ref) |  |
| **TC+CC** | 222/130 | 1.26 (0.96-1.64) | 0.09 | 1.55 (1.13-2.12) | 0.006 | 1.52 (1.09-2.10) | 0.012 |
| **Recessive** |  |  |  |  |  |  |  |
| **TT+TC** | 718/357 | 1.0 (ref) |  | 1.0 (ref) |  | 1.0 (ref) |  |
| **CC** | 21/14 | 1.34 (0.67-2.67) | 0.40 | 1.35 (0.60-3.02) | 0.47 | 1.27 (0.55-2.94) | 0.58 |
| **rs947895** |  |  |  |  |  |  |  |
| **CC** | 523/247 | 1.0 (ref) |  | 1.0 (ref) |  | 1.0 (ref) |  |
| **CA** | 174/107 | 1.30 (0.98-1.73) | 0.07 | 1.60 (1.14-2.23) | 0.006 | 1.58 (1.12-2.25) | 0.010 |
| **AA** | 20/11 | 1.17 (0.55-2.47) | 0.69 | 1.31 (0.55-3.08) | 0.54 | 1.25 (0.51-3.06) | 0.63 |
| **Dominant** |  |  |  |  |  |  |  |
| **CC** | 523/247 | 1.0 (ref) |  | 1.0 (ref) |  | 1.0 (ref) |  |
| **CA+AA** | 194/118 | 1.29 (0.98-1.69) | 0.07 | 1.56 (1.13-2.16) | 0.007 | 1.55 (1.10-2.16) | 0.011 |
| **Recessive** |  |  |  |  |  |  |  |
| **CC+CA** | 697/354 | 1.0 (ref) |  | 1.0 (ref) |  | 1.0 (ref) |  |
| **AA** | 20/11 | 1.08 (0.51-2.29) | 0.83 | 1.15 (0.49-2.70) | 0.75 | 1.10 (0.45-2.67) | 0.84 |

OR: odds ratio, 95% CI: 95% confidence interval. ^a^ *P*-values were calculated using the χ^2^ test. Model I: crude OR; Model II: age (continuous), BMI (<25 kg/m^2^ or ≥25 kg/m^2^), education level (less than college or college and higher), income (<200, 200- <400 or ≥400), physical activity (yes or no), smoking status (current, ex- or non-smoker), first-degree family history of GC (yes or no), and total energy intake; Model III: additionally, adjusted for *H. pylori* infection (positive or negative).

**Table S6. Association between dietary ORAC intake and GC risk by *GSTP1* SNPs**

| ***GSTP1* SNPs** | | **No. Controls/Cases** | | **Model I OR (95% CI)** | | **Model II OR (95% CI)** | | **Model III OR (95% CI)** | |
| --- | --- | --- | --- | --- | --- | --- | --- | --- | --- |
| **rs749174 (dominant)** | | **GG** | **GA+AA** | **GG** | **GA+AA** | **GG** | **GA+AA** | **GG** | **GA+AA** |
| H-ORAC (μmol TE/day) | T1 (<2655.88) | 175/122 | 61/65 | 1.0 (ref) | 1.0 (ref) | 1.0 (ref) | 1.0 (ref) | 1.0 (ref) | 1.0 (ref) |
|  | T2 (2655.88-4759.10) | 176/73 | 64/31 | 0.60 (0.42-0.85) | 0.46 (0.26-0.79) | 0.73 (0.48-1.12) | 0.33 (0.16-0.68) | 0.75 (0.48-1.17) | 0.39 (0.19-0.82) |
|  | T3 (>4759.10) | 172/52 | 69/22 | 0.43 (0.30-0.64) | 0.30 (0.17-0.54) | 0.63 (0.40-0.99) | 0.31 (0.15-0.64) | 0.65 (0.40-1.04) | 0.36 (0.17-0.77) |
|  | *P* for trend |  |  | < 0.001 | < 0.001 | 0.054 | 0.003 | 0.080 | 0.013 |
| L-ORAC (μmol TE/day) | T1 (<140.01) | 170/115 | 65/49 | 1.0 (ref) | 1.0 (ref) | 1.0 (ref) | 1.0 (ref) | 1.0 (ref) | 1.0 (ref) |
|  | T2 (140.01-215.68) | 175/76 | 66/44 | 0.64 (0.45-0.92) | 0.88 (0.52-1.51) | 0.67 (0.44-1.02) | 0.80 (0.41-1.53) | 0.65 (0.42-1.02) | 0.66 (0.33-1.33) |
|  | T3 (>215.68) | 178/56 | 63/25 | 0.47 (0.32-0.68) | 0.53 (0.29-0.95) | 0.58 (0.37-0.91) | 0.80 (0.39-1.64) | 0.58 (0.37-0.93) | 0.86 (0.40-1.86) |
|  | *P* for trend |  |  | < 0.001 | 0.035 | 0.015 | 0.524 | 0.020 | 0.642 |
| TPs  (mg GAE/day) | T1 (<230.58) | 170/127 | 67/65 | 1.0 (ref) | 1.0 (ref) | 1.0 (ref) | 1.0 (ref) | 1.0 (ref) | 1.0 (ref) |
|  | T2 (230.58-445.51) | 176/69 | 62/33 | 0.53 (0.37-0.75) | 0.55 (0.32-0.94) | 0.65 (0.43-0.99) | 0.47 (0.24-0.94) | 0.68 (0.44-1.06) | 0.45 (0.22-0.92) |
|  | T3 (>445.51) | 177/51 | 65/20 | 0.39 (0.26-0.57) | 0.32 (0.17-0.58) | 0.61 (0.38-0.97) | 0.34 (0.16-0.71) | 0.64 (0.39-1.03) | 0.38 (0.17-0.83) |
|  | *P* for trend |  |  | < 0.001 | < 0.001 | 0.045 | 0.005 | 0.083 | 0.019 |
| **rs4891 (dominant)** | | **TT** | **TC+CC** | **TT** | **TC+CC** | **TT** | **TC+CC** | **TT** | **TC+CC** |
| H-ORAC (μmol TE/day) | T1 (<2655.88) | 174/117 | 70/74 | 1.0 (ref) | 1.0 (ref) | 1.0 (ref) | 1.0 (ref) | 1.0 (ref) | 1.0 (ref) |
|  | T2 (2655.88-4759.10) | 171/72 | 73/33 | 0.63 (0.44-0.90) | 0.43 (0.25-0.72) | 0.79 (0.62-1.22) | 0.36 (0.18-0.70) | 0.81 (0.52-1.28) | 0.42 (0.21-0.84) |
|  | T3 (>4759.10) | 172/52 | 79/23 | 0.45 (0.31-0.66) | 0.28 (0.16-0.49) | 0.67 (0.42-1.06) | 0.32 (0.16-0.66) | 0.69 (0.43-1.11) | 0.37 (0.18-0.75) |
|  | *P* for trend |  |  | < 0.001 | < 0.001 | 0.094 | 0.002 | 0.134 | 0.008 |
| L-ORAC (μmol TE/day) | T1 (<140.01) | 168/111 | 75/56 | 1.0 (ref) | 1.0 (ref) | 1.0 (ref) | 1.0 (ref) | 1.0 (ref) | 1.0 (ref) |
|  | T2 (140.01-215.68) | 174/74 | 75/46 | 0.64 (0.45-0.93) | 0.82 (0.50-1.36) | 0.68 (0.45-1.05) | 0.71 (0.38-1.33) | 0.67 (0.43-1.05) | 0.60 (0.31-1.15) |
|  | T3 (>215.68) | 175/56 | 72/28 | 0.48 (0.33-0.71) | 0.52 (0.30-0.91) | 0.61 (0.39-0.96) | 0.79 (0.40-1.58) | 0.61 (0.38-0.98) | 0.83 (0.40-1.70) |
|  | *P* for trend |  |  | < 0.001 | 0.022 | 0.028 | 0.479 | 0.036 | 0.534 |
| TPs  (mg GAE/day) | T1 (<230.58) | 169/123 | 78/72 | 1.0 (ref) | 1.0 (ref) | 1.0 (ref) | 1.0 (ref) | 1.0 (ref) | 1.0 (ref) |
|  | T2 (230.58-445.51) | 172/67 | 70/37 | 0.54 (0.37-0.77) | 0.57 (0.34-0.96) | 0.68 (0.44-1.04) | 0.54 (0.28-1.04) | 0.72 (0.46-1.13) | 0.55 (0.28-1.07) |
|  | T3 (>445.51) | 176/51 | 74/21 | 0.40 (0.27-0.59) | 0.31 (0.17-0.55) | 0.64 (0.40-1.02) | 0.37 (0.18-0.74) | 0.68 (0.42-1.10) | 0.40 (0.19-0.84) |
|  | *P* for trend |  |  | < 0.001 | < 0.001 | 0.073 | 0.006 | 0.136 | 0.018 |
| **rs947895 (dominant)** | | **CC** | **CA+AA** | **CC** | **CA+AA** | **CC** | **CA+AA** | **CC** | **CA+AA** |
| H-ORAC  (μmol TE/day) | T1 (<2655.88) | 175/122 | 61/65 | 1.0 (ref) | 1.0 (ref) | 1.0 (ref) | 1.0 (ref) | 1.0 (ref) | 1.0 (ref) |
|  | T2 (2655.88-4759.10) | 175/73 | 64/31 | 0.60 (0.42-0.86) | 0.46 (0.26-0.79) | 0.75 (0.49-1.15) | 0.33 (0.16-0.68) | 0.77 (0.49-1.20) | 0.39 (0.19-0.82) |
|  | T3 (>4759.10) | 173/52 | 69/22 | 0.43 (0.29-0.64) | 0.30 (0.17-0.54) | 0.63 (0.40-1.00) | 0.31 (0.15-0.64) | 0.65 (0.41-1.05) | 0.36 (0.17-0.78) |
|  | *P* for trend |  |  | < 0.001 | < 0.001 | 0.055 | 0.003 | 0.084 | 0.013 |
| L-ORAC  (μmol TE/day) | T1 (<140.01) | 170/115 | 65/49 | 1.0 (ref) | 1.0 (ref) | 1.0 (ref) | 1.0 (ref) | 1.0 (ref) | 1.0 (ref) |
|  | T2 (140.01-215.68) | 175/76 | 66/44 | 0.64 (0.45-0.92) | 0.88 (0.52-1.51) | 0.68 (0.44-1.03) | 0.80 (0.41-1.53) | 0.67 (0.43-1.03) | 0.66 (0.33-1.33) |
|  | T3 (>215.68) | 178/56 | 63/25 | 0.47 (0.32-0.68) | 0.53 (0.29-0.95) | 0.58 (0.37-0.91) | 0.80 (0.39-1.64) | 0.58 (0.37-0.93) | 0.86 (0.40-1.86) |
|  | *P* for trend |  |  | < 0.001 | 0.035 | 0.016 | 0.524 | 0.021 | 0.642 |
| TPs  (mg GAE/day) | T1 (<230.58) | 170/127 | 67/65 | 1.0 (ref) | 1.0 (ref) | 1.0 (ref) | 1.0 (ref) | 1.0 (ref) | 1.0 (ref) |
|  | T2 (230.58-445.51) | 175/69 | 62/33 | 0.53 (0.37-0.76) | 0.55 (0.32-0.94) | 0.66 (0.43-1.01) | 0.47 (0.24-0.94) | 0.70 (0.45-1.09) | 0.45 (0.22-0.92) |
|  | T3 (>445.51) | 178/51 | 65/20 | 0.38 (0.26-0.57) | 0.32 (0.17-0.58) | 0.61 (0.38-0.97) | 0.34 (0.16-0.71) | 0.64 (0.40-1.04) | 0.38 (0.17-0.83) |
|  | *P* for trend |  |  | < 0.001 | < 0.001 | 0.046 | 0.005 | 0.087 | 0.019 |

ORAC: oxygen radical absorbance capacity, H-ORAC: hydrophilic oxygen radical absorbance capacity, L-ORAC: lipophilic oxygen radical absorbance capacity, TPs: total phenolics, TE: Trolox equivalents, GAE: gallic acid equivalents, T: tertile, OR: odds ratio, 95% CI: 95% confidence interval. Model I: crude OR; Model II: age (continuous), BMI (<25 kg/m^2^ or ≥25 kg/m^2^), education level (less than college or college and higher), income (<200, 200- <400 or ≥400), physical activity (yes or no), smoking status (current, ex- or non-smoker), first-degree family history of GC (yes or no), and total energy intake; Model III: additionally, adjusted for *H. pylori* infection (positive or negative).

**Table S7. Interaction between dietary ORAC intake and GC risk by rs1871042 polymorphism of *GSTP1* gene**

| ***GSTP1*** | | **No. Controls/Cases** | | **Model I OR (95% CI)** | | **Model II OR (95% CI)** | | **Model III OR (95% CI)** | |
| --- | --- | --- | --- | --- | --- | --- | --- | --- | --- |
| **rs1871042 (dominant)** | | **CC** | **CT+TT** | **CC** | **CT+TT** | **CC** | **CT+TT** | **CC** | **CT+TT** |
| H-ORAC (μmolTE/day) | Low (<3638.60) | 264/168 | 94/85 | 1.0 (ref) | 1.42 (1.00-2.02) | 1.0 (ref) | **1.66 (1.12-2.47)** | 1.0 (ref) | **1.64 (1.08-2.49)** |
|  | High (≥3638.60) | 259/79 | 100/33 | 0.48 (0.35-0.66) | 0.52 (0.33-0.80) | **0.57 (0.40-0.81)** | 0.75 (0.46-1.21) | **0.63 (0.44-0.91)** | 0.76 (0.46-1.25) |
|  | *P* for interaction |  |  | 0.36 | | 0.46 | | 0.36 | |
| L-ORAC (μmolTE/day) | Low (<176.09) | 265/160 | 94/68 | 1.0 (ref) | 1.20 (0.83-1.73) | 1.0 (ref) | 1.38 (0.91-2.09) | 1.0 (ref) | 1.31 (0.85-2.02) |
|  | High (≥176.09) | 258/87 | 100/50 | 0.56 (0.41-0.76) | 0.83 (0.56-1.23) | **0.60 (0.43-0.85)** | 1.07 (0.69-1.66) | **0.60 (0.42-0.87)** | 1.05 (0.66-1.67) |
|  | *P* for interaction |  |  | 0.45 | | 0.43 | | 0.38 | |
| TPs (mgGAE/day) | Low (<320.65) | 263/167 | 96/85 | 1.0 (ref) | 1.39 (0.98-1.98) | 1.0 (ref) | **1.71 (1.15-2.54)** | 1.0 (ref) | **1.67 (1.11-2.53)** |
|  | High (≥320.65) | 260/80 | 98/33 | 0.49 (0.35-0.67) | 0.53 (0.34-0.82) | **0.63 (0.44-0.90)** | 0.78 (0.48-1.26) | **0.68 (0.47-0.99)** | 0.79 (0.47-1.31) |
|  | *P* for interaction |  |  | 0.42 | | 0.31 | | 0.28 | |

ORAC: oxygen radical absorbance capacity, H-ORAC: hydrophilic oxygen radical absorbance capacity, L-ORAC: lipophilic oxygen radical absorbance capacity, TPs: total phenolics, TE: Trolox equivalents, GAE: gallic acid equivalents, OR: odds ratio, 95% CI: 95% confidence interval. Dietary ORAC intake was divided into two groups (low vs. high) according to the median level of the intake of the controls. Model I: crude OR; Model II: age (continuous), BMI (<25 kg/m^2^ or ≥25 kg/m^2^), education level (less than college or college and higher), income (<200, 200- <400 or ≥400), physical activity (yes or no), smoking status (current, ex- or non-smoker), first-degree family history of GC (yes or no), and total energy intake; Model III: additionally, adjusted for *H. pylori* infection (positive or negative).
